# Seroprevalence of SARS-CoV-2 antibodies among homeless people living rough, in shelters and squats: A large population-based study in France

**DOI:** 10.1371/journal.pone.0255498

**Published:** 2021-09-15

**Authors:** Sandrine Loubiere, Elisabetta Monfardini, Camille Allaria, Marine Mosnier, Agathe Allibert, Laetitia Ninove, Thomas Bosetti, Cyril Farnarier, Ilyes Hamouda, Pascal Auquier, Emilie Mosnier, Aurélie Tinland

**Affiliations:** 1 Department of Research and Innovation, Assistance Publique Hôpitaux de Marseille - APHM, Marseille, France; 2 CEReSS: Health Service Research and Quality of Life Center, Aix Marseille University, Marseille, France; 3 Department of Psychiatry, Sainte-Marguerite University Hospital, APHM, Marseille, France; 4 INSERM, U1028, CNRS UMR5292, Lyon Neuroscience Research Center, ImpAct Team, Bron, France; 5 LAMES – Laboratoire Méditerranéen de Sociologie, UMR 7305, MMSH – Maison Méditerranéenne des Sciences de l’Homme, Aix-en-Provence, France; 6 Médecins du Monde, Marseille, France; 7 Unité des Virus Emergents (UVE), Aix-Marseille Univ – IRD 190 – Inserm 1207 –IHU Méditerranée Infection, Marseille, France; 8 Projet ASSAb – Accès aux Soins pour les Personnes Sans Abri, Hôpital Européen, Marseille, France; 9 LaSSA – Laboratoire de Sciences Sociales Appliquées, Marseille, France; 10 INSERM, IRD, SESSTIM, Sciences Economiques & Sociales de la Santé & Traitement de l’Information Médicale, Aix Marseille University, Marseille, France; Centers for Disease Control and Prevention, UNITED STATES

## Abstract

**Background:**

Overcrowded housing, as well as inadequate sanitary conditions, contribute to making homeless people particularly vulnerable to the SARS-CoV-2 infection. We aimed to assess the seroprevalence of the SARS-CoV-2 infection among people experiencing homelessness on a large city-wide scale in Marseille, France, taking into account different types of accommodation.

**Methods:**

A consortium of outreach teams in 48 different locations including streets, slums, squats, emergency or transitional shelters and drop-in centres participated in the inclusion process. All participants consented to have a validated rapid antibody assay for immunoglobulins M (IgM) and G (IgG) and to answer a questionnaire on medical health conditions, comorbidities, and previous COVID-19 symptoms. Information on their housing conditions since the COVID-19 crisis was also collected from the participants.

**Results:**

From June 01 to August 05, 2020, 1,156 homeless participants were enrolled in the study and tested. The overall seroprevalence of SARS-CoV-2 IgG/IgM antibodies was 5.6% (95%CI 2.3–7.0), ranging from 2.2% in people living on the streets to 8.1% in people living in emergency shelters (P = 0.009). Around one third of the seropositive participants reported COVID-19 symptoms. Compared to the general population in Marseille (3.6%), the homeless population living in the same urban area experienced a significantly increased risk of SARS-CoV-2 infection (|z| = 3.65 > 1.96).

**Conclusion:**

These findings highlight the need for regular screening among the homeless to prevent clustering in overcrowded or inadequate accommodations. It is also necessary to provide essential resources to keep homeless people healthy, the vast majority of whom have cumulative risk factors for SARS-CoV-2 infection.

## Introduction

In 2016, nearly 5.3 million individuals (i.e., 2% of the population) had been without shelter, or in emergency or temporary accommodation at least once in their lifetime across Europe [1]. This surpassed previous estimates by far, which ranged from 0.1% to 0.3% across European countries [2, 3]. The recent increase in the number of people experiencing homelessness is likely to further increase due to the current severe acute respiratory syndrome coronavirus 2 (SARS-CoV-2) crisis [4–7].

Homeless people should be particularly vulnerable to SARS-CoV-2 infection: on the one hand, they cumulate risk factors for SARS-CoV-2 contamination, such as living in overcrowded or inadequate accommodation (squats, slums, or shared rooms in shelters), or having frequent contact with people through community aid services (food distribution or mobile health facilities); on the other hand, they are at increased risk for severe SARS-CoV-2 disease, being exposed to a high prevalence of comorbidities, particularly respiratory and heart diseases, in addition to an ageing issue [8–11].

Previous literature has pointed out the challenge of providing care for the homeless during the SARS-CoV-2 pandemic [9] and reported explorations of SARS-CoV-2 prevalence using virological tests in one, three or five shelters [12–14], particularly in U.S. settings. Such explorations provided interesting clues on the environmental factors favouring the spread of SARS-CoV-2 transmission, such as moves between homeless accommodations, overcrowded and congregate settings, where physical distancing was challenging. To our knowledge, there is a lack of European data, as well as a lack of systematic assessments of the impact of the SARS-CoV-2 infection in the homeless population taken as a whole, on a city-wide scale rather than in specific living settings where public health teams responded to clusters.

In the present cross-sectional study, which is part of a broader population-based cohort study, named COVID_Homeless, on morbidity and mortality due to SARS-CoV-2 among the homeless population, our aims were: 1) to assess the seroprevalence of SARS-CoV-2 during the first wave of SARS-CoV-2 outbreak within the homeless population living in Marseille, the second most populated French city and one of its poorest, which was also the second zone in France with active circulation of the virus at the time of the study period [15]; 2) to compare seroprevalence estimates according to living conditions, sociodemographic and medical conditions in order to assess correlates of seroprevalence.

## Materials and methods

### Study design and study participants

The present cross-sectional seroprevalence study is part of a large prospective population-based cohort survey of homeless people living in Marseille, France (“COVID-Homeless”). Participants were enrolled between June 1 and August 5, 2020 from 48 different homeless spots in the city, including streets, slums, squats, emergency and transitional shelters, and drop-in centres. Eligible individuals were aged over 18 and lived in the following typology of homelessness (according to the ETHOS—European typology for homelessness and housing exclusion, which is a framework definition for policy and practice purposes stated at the European level) [16]: i) living rough (ETHOS1), ii) living in emergency accommodations (emergency shelters and hotels) (ETHOS2); iii) living in transitional accommodations for the homeless (ETHOS3); and iv) living in insecure accommodations (i.e., illegal occupation of lands, squat/slum or temporarily with family/friends) (ETHOS8).

### Sample size and sampling procedure

**Fig 1:**
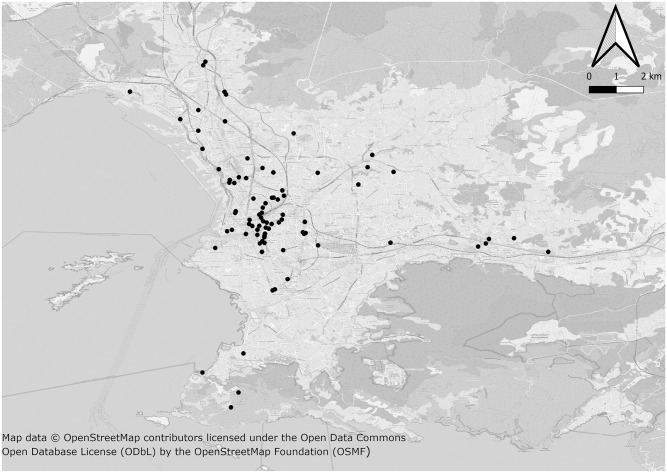
Mapping locations of homeless people participating in the study, Marseille, France. A base map was extracted from OpenStreetMap (http://www.openstreetmap.org) [18]. OpenStreetMap is open data, licensed under the Open Data Commons Open Database License (ODbL) by the OpenStreetMap Foundation (OSMF). The map was generated using QGIS 3.16 software (GNU licence) [19].

The 48 homeless settings were identified in partnership with all of the outreach teams from public health and social services and community partners working in Marseille, France, and who participated in the enrolment phase (Fig 1). This study considered outreach teams’ registers for homeless facilities as well as users’ registers for each enrolled facility over a given period. There was no initial sampling since the purpose of the COVID-Homeless study was to be exhaustive. Although comprehensive homelessness prevalence data for Marseille are still lacking, we used data from the local Integrated Reception and Orientation Service (IROS–SIAO in French) for emergency and transitional accommodations, and NGO estimations for slums/squats and streets: 775 people living in emergency shelters, 300 in hotels, 443 in transitional shelters, 840 in squats/slums and 400 as rough sleepers. These data did not include dispersed accommodation facilities (10 settings), family shelters (3 settings) or any children from squats since children were excluded from the study (i.e. an Ethics Board’s decision). Given the potential refusal of homeless people living in enrolled facilities, we calculated a minimum sample size to be reached to achieve appropriate SARS-CoV-2 seroprevalence estimates [17]. A sample size of 430 from a population of 2,800 gave a two-sided 95% confidence interval with a precision (half-width) of 0.02 (when the actual seroprevalence is near 0.05).

### Ethics statement

This study was approved by the local Research Ethics committee on May 28, 2020 (number 44–20). Participants signed a consent form to participate after having received information on the study’s purposes, intended data use, and being ensured anonymity. ClinicalTrials.gov ID: NCT04408131 on May 29, 2020.

### Data collection

All participants had a rapid serological test for SARS-CoV-2 IgM/IgG antibodies. A face-to-face questionnaire was also filled in using Redcap software (www.project-redcap.org) and including demographic characteristics, type of homelessness and housing conditions since the SARS-CoV-2 outbreak, comorbidities (diabetes, cancer, chronic respiratory disease, cardiovascular disease, hypertension, immunodeficiency, or chronic kidney disease) and past or present symptoms of COVID-19. The questionnaire is available in the S1 File.

### Serology testing

To assess the seroprevalence rates of SARS-CoV-2, we used validated serological tests manufactured by the French company Biosynex (Biosynex COVID-19 BSS) that provided immunoglobulins M (IgM) and G (IgG) results within 10 minutes. In the validation phase, the serological assay showed sensitivity of 91.8% (95%CI: 83.8%-96.6%), and specificity of 99.2% (95%CI: 97.7%-99.8%) for IgM antibodies (based on 456 samples) and 100% (95%CI: 96.1%-100%) and 99.5% (95%CI: 98.1%-99.9%), respectively, for IgG (based on 446 samples) (www.biosynex.com). An independent validation study was carried out by the National Reference Centre (CNR Institute Pasteur and CNR associated laboratory of the Hospices Civils de Lyon). The specific steps in the validation process are explicitly explained in the procedural guidelines as follows: 1) Constitution of the panel of sera: For specificity evaluation, a minimum of 50 pre-pandemic sera were considered, for sensitivity evaluation, at least 50 sera from patients infected with COVID-19 (i.e., with a positive RT-PCR and hospitalized) at different times after the onset of symptoms, were considered; 2) Performance evaluation with a panel of EC-approved diagnostic medical devices, *in vitro*: tests from the same production batch were used; 3) Interpretation of results: the control strip was validated for each serum; if not, the point was excluded as in the case of a defective cassette, and all exclusions were noted in a report. Dubious results were counted as positive, and mentioned as a comment in the report if this concerned more than 20% of the positive results. This evaluation gave similar results to those of Biosynex.

### Data management and data quality control

A pilot study was conducted with a sample of 10 individuals to assess the length of the questionnaire and its intelligibility (face validity). Then, the survey questionnaire was translated into the targeted native languages. Throughout data collection, the data manager checked the content of the main outcomes (date of inclusion, results of serology testing, place of living) in order to control for missing data and to resolve this immediately. To check for duplicates at inclusion, we performed a careful prospective check of each inclusion. In addition, at the end of the study, a quality control of each inclusion was conducted and the people in charge of monitoring and control looked for a similar date of birth, name and surname. Any duplicates were excluded from the analysis.

### Statistical analysis

Descriptive analyses were presented as frequencies and percentages for categorical variables, and as means and standard deviations for quantitative variables. In this study, SARS-CoV-2 seroprevalence was defined as the proportion of individuals who had a positive result in the IgG or IgM band of the rapid serological test. Seroprevalence estimates were given as proportions with 95% confidence intervals (95%CI). Bootstrap resampling approach with a set of 1,000 samples was used to create confidence intervals, accounting for variability in the sensitivity and specificity of the serological assay. We compared the proportion of seropositive cases to demographic characteristics, living conditions, health characteristics and comorbidities, using Chi-2 test (or Fisher’s exact test when one or more expected cell counts in the 2x2 cross-table were under 5) for qualitative characteristics and Student’s t-test (or Mann-Whitney test depending on the variable distribution) for quantitative measures. Wilcoxon rank-sum tests were used to compare the proportion of positive SARS-CoV-2 tests among homeless typology, demographic groups, symptoms and comorbidities. Statistical analysis was performed using R version 3.6.0 software [20].

## Results

**Fig 2:**
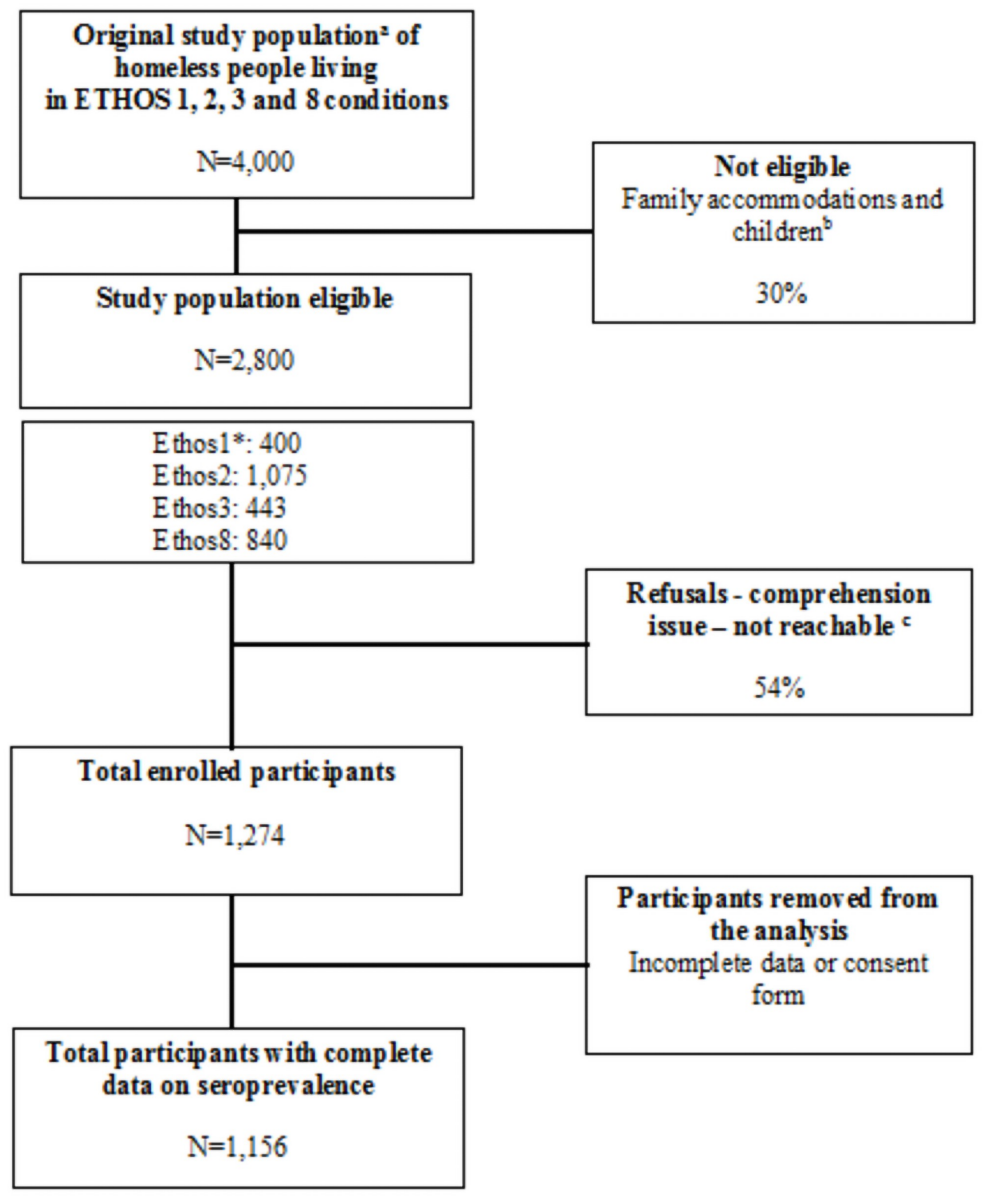
Flow chart of the SARS-CoV-2 seroprevalence study among people experiencing homelessness, Marseille, France. a: Although comprehensive homelessness prevalence data for Marseille are still lacking, we used data from local Integrated Reception and Orientation Service (IROS–SIAO in French) for emergency and transitional accommodations, and estimations of NGOs for slums/squats and streets; b: estimations of NGOs at around 30% of squat inhabitants; c: Reasons for not being included in the study were difficult to distinguished as most of people living in squats or streets cumulated the three main reasons (refusals, comprehension issue or unreachable at the time of the study took place); *: ETHOS: the European typology for homelessness and housing exclusion; ETHOS1: living rough; ETHOS2: living in emergency accommodations (emergency shelters and hotels); ETHOS3: living in transitional accommodations for homeless persons; and ETHOS8: living in insecure accommodations (i.e., illegal occupation of lands, squat/slum or temporarily with family/friends).

Between June 1 and August 5, 2020, we enrolled 1,274 individuals. After quality control, 118 individuals presented issues with their data (i.e., missing data for the primary outcome and/or a missing consent form). A total of 1,156 (90.7%) participants were eligible for the study and included in the analysis (see Fig 2). The mean age was 40.2 years (standard deviation: 14.3) with 65 (5.4%) participants over 65 years old. The majority of respondents were men (n = 824, 71.3%), with a lower secondary education level or no academic achievement (960, 83.1%) and with health insurance (794, 70.3%), this included 197 (24.8%) people covered by state health insurance (Table 1). A large majority of respondents were foreign (940, 81.3%) and were born abroad: 485 (42.1%) from African countries, 187 (16.2%) from European Union (EU) countries, 203 (17.6%) from non-EU European countries and 65 (5.4%) were from countries outside Africa and Europe. A maximum of 159 (14%) reported that they were working at the time of the survey (legal or illegal employment). A total of 348 (30.2%) participants were living in emergency shelters, 195 (16.9%) in hotel rooms, 192 (16.7%), in transitional shelters, 329 (28.5%) in squats/slums and 89 (7.2%) in the street (Table 1). One third (32.4%) reported long-term homelessness (>5 years) and one half (53.3%) reported having at least one comorbidity.

**Table 1:** Sociodemographic characteristics of the study population (n = 1,156).

Sociodemographic characteristics	n (%) or mean (SD)	
Gender		
Men	824	(71.3%)
Women	332	(28.7%)
Age, year	40.2	(14.3)
French Nationality ^a^	208	(18.1%)
Country of Birth^$^^,^^§^		
France	216	(18.7%)
European union	187	(16.2%)
Non-EU Europe	203	(17.5%)
Africa	485	(42.0%)
Other	65	(5.6%)
Education level		
No academic achievement	529	(45.8%)
Lower secondary	431	(37.3%)
Upper secondary or vocational	123	(10.6%)
Not known or missing	73	(6.3%)
Living with someone ^b^	517	(45.2%)
Health insurance ^c^^,^^$^	794	(70.3%)
State health insurance or other^$^		
State health insurance	197	(17.4%)
Other	597	(52.9%)
None	335	(29.7%)
Having financial resources		
No	497	(43.0%)
Yes	622	(53.8%)
Missing	37	(3.2%)
Having a working situation		
No	960	(83.1%)
Yes	159	(13.7%)
Missing	37	(3.2%)
**Living conditions**	**n**	(%)
Total duration of homelessness		
<3 months	83	(7.2%)
3 to 12 months	228	(19.7%)
1 to 5 years	426	(36.9%)
>5 years	374	(32.4%)
Missing data	45	(3.9%)
Typology ETHOS* at baseline ^$^		
ETHOS 1: street	89	(7.7%)
ETHOS 2: emergency shelters	348	(30.2%)
ETHOS2: hotel rooms	195	(16.9%)
ETHOS 3: transitional shelters	192	(16.7%)
ETHOS 8: squats, slums	329	(28.5%)
Type of accommodation		
Private room or area	504	(44.3%)
Shared room or area	634	(55.7%)
Change of accommodation during SARS_COV2 crisis		
No	686	(59.3%)
Yes	428	-37%
Missing	42	(3.6%)
**Health characteristics**	**n**	(%)
Tobacco consumption		
No	463	(40.1%)
Yes	608	(52.6%)
Missing	85	(7.4%)
Alcohol consumption		
none	766	(66.3%)
<3 glasses	135	(11.7%)
> = 3 or more glasses	128	(11.1%)
Missing	127	-11%
Illegal substance consumption		
No	850	(73.5%)
Yes	200	(17.3%)
Missing	106	(9.2%)
Comorbidities ^d^		
Having at least one comorbidity (% yes)	617	(53.3%)
Number of comorbidities	1	(1.3)
Psychiatric and addiction comorbidities (% yes)	272	(23.5%)
Existence of risk factors for severe SARS-CoV-2 disease		
Obesity (% yes)	74	(6.5%)
Diabetes (% yes)	87	(7.7%)
Cancer (% yes)	23	(2.0%)
Chronic Respiratory Pathology (% yes)	92	(8.5%)
Cardiovascular Pathology (% yes)	147	(13.5%)
Chronic renal failure (% yes)	22	(2.0%)

^a^: the proportion of ’No French nationality’ can be deduced;

^b^: the proportion of ’Single individual’ can be deduced;

^c^: the proportion of ’No health insurance’ can be deduced;

^d^: the proportion of ‘no comorbidities’ can be deduced.

*ETHOS: the European typology for homelessness and housing exclusion. SD: standard deviation.

^$^: missing data were less than 3% and were not reported.

^§^: “European Union” countries: Belgium, Bulgaria, Germany, Hungary, Italy, Poland, Portugal, Romania, Czech Republic, Slovakia, and Spain. “Non-EU European” countries: Albania, Armenia, Bosnia, Croatia, Moldavia, Montenegro, Serbia, Russia including Chechenia, and Ukraine.

Table 2 shows the seroprevalence of SARS-CoV-2 in the study population. In this study, 58 out of 1,156 were positive for SARS-CoV-2 IgG antibodies (5.0%); 24 out of 1,156 were positive for SARS-CoV-2 IgM antibodies (2.1%); 17 (1.5%) were positive for both SARS-CoV-2 IgG and IgM antibodies. Overall, seroprevalence was 5.62% (95%CI: 2.90–7.21).

**Table 2:** Rapid serological testing results for SARS-CoV-2 (N = 1,156).

	Seronegative cases	Seropositive cases	95%CI ^$^
	n (%)	n (%)	
***Rapid serological testing***			
IgM	1132 (97.92%)	24 (2.08%)	(0.27–2.26)
IgG	1098 (94.98%)	58 (5.02%)	(3.13–5.20)
Seroprevalence	1091 (94.38%)	65 (5.62%)	(2.90–7.21)

95% IC: 95%confidence interval; IgM/IgG: immunoglobulins M and G against SARS-CoV-2.

^$^: Bootstrap resampling approach with a set of 1,000 samples was used to create 95% confidence intervals.

Seroprevalence was not statistically different between men and women, between people living with someone and living alone, between those working and those not working (Table 3). No differences were found according to age, country of birth or level of education. Seroprevalence estimates were significantly different between ETHOS categories: 8.10% (95%CI; 5.89–10.63) in ETHOS 2 (i.e. emergency shelters and hotels), 3.95% (95%CI: 2.12–6.66) in ETHOS 8 (i.e. squats/slums), 3.12% (95%CI: 1.16–6.68) in ETHOS 3 (i.e. transitional shelters) and 2.25% (95%CI: 0.27–7.88) in ETHOS 1 (i.e. rough sleepers) (P = 0.009). Among homeless participants tested positive for SARS-CoV-2, 56.9% (37/65) had spent more than one month in emergency shelters, compared to 29.5% (313/1091) of participants with negative tests (P < 0.001).

**Table 3:** Seroprevalence of SARS-CoV-2 according to demographic characteristics, living conditions, health characteristics and comorbidities (N = 1,156).

	Seronegative cases		Seropositive cases		95%IC	p
*Demographic characteristics*	N	(%)	N	(%)		
Gender						
Men	780	(94.66%)	44	(5.34%)	(3.91–7.1)	0.572
Women	311	(93.67%)	21	(6.33%)	(3.96–9.51)	
Age, year	40.1	(14.3)	42.2	(14.5)		0.271
French Nationality						
No	882	(93.73%)	59	(6.27%)	(4.81–8.01)	0.067
Yes	202	(97.12%)	6	(2.88%)	(1.07–6.17)	
Country of Birth^§^						
France	207	(95.83%)	9	(4.17%)	(1.92–7.76)	0.382
European Union	179	(95.7%)	8	(4.3%)	(1.86–8.26)	
Non-EU Europe	194	(95.6%)	9	(4.4%)	(2.05–8.25)	
Africa	450	(92.78%)	35	(7.22%)	(5.08–9.89)	
Other	58	(93.55%)	4	(6.45%)	(1.79–15.7)	
Education level						
No academic achievement	504	(94.92%)	25	(4.71%)	(3.07–6.87)	0.420
Lower secondary	402	(91.78%)	29	(6.62%)	(4.48–9.37)	
Upper secondary or vocational	117	(95.12%)	6	(4.88%)	(1.81–10.32)	
Not known or missing	68	(86.08%)	5	(6.33%)	(2.09–14.16)	
Living with someone						
No	592	(94.27%)	36	(5.73%)	(4.05–7.85)	0.795
Yes	490	(94.78%)	27	(5.22%)	(3.47–7.51)	
Health insurance						
No	322	(96.12%)	13	(3.88%)	(2.08–6.54)	0.122
Yes	743	(93.58%)	51	(6.42%)	(4.82–8.36)	
State health insurance or other						
State health insurance	184	(93.4%)	13	(6.6%)	(3.56–11.02)	0.227
Other	559	(93.63%)	38	(6.37%)	(4.54–8.63)	
None	322	(96.12%)	13	(3.88%)	(2.08–6.54)	
Having financial resources						
No	463	(93.15%)	34	(6.84%)	(4.78–9.42)	0.199
Yes	591	(95.01%)	31	(4.98%)	(3.41–6.84)	
Having work						
No	899	(93.64%)	61	(6.35%)	(4.89–8.08)	0.065
Yes	155	(97.48%)	4	(2.51%)	(0.68–6.31)	
***Living conditions***						
Total duration of homelessness						
<3 months	79	(92.94%)	4	(4.71%)	(1.3–11.61)	0.787
3 to 12 months	218	(94.78%)	10	(4.35%)	(2.1–7.85)	
1 to 5 years	399	(92.79%)	27	(6.28%)	(4.18–9)	
>5 years	353	(93.88%)	21	(5.59%)	(3.49–8.41)	
Typology ETHOS* at baseline						
ETHOS 1: street	87	(97.75%)	2	(2.25%)	(0.27–7.88)	**0.009**
ETHOS2: emergency shelters and hotels	499	(91.9%)	44	(8.1%)	(5.89–10.63)	
ETHOS 3: transitional shelters	186	(96.88%)	6	(3.12%)	(1.16–6.68)	
ETHOS 8: squats, slums	316	(96.05%)	13	(3.95%)	(2.12–6.66)	
Type of accommodation						
Private room or area	477	(93.9%)	27	(5.31%)	(3.53–7.64)	0.896
Shared room or area	598	(94.17%)	36	(5.67%)	(4–7.76)	
Time spent in emergency shelters						
Less than one month	747	(96.39%)	28	(3.61%)	(2.41–5.18)	**<0.001**
More than one month	313	(89.43%)	37	(10.57%)	(7.55–14.28)	
Contacts per day, number	9.2	(12.2)	6.1	(5.9)		**0.001**
Change of accommodation during SARS-CoV-2 crisis						
No	655	(95.48%)	31	(4.52%)	(3.09–6.35)	0.08
Yes	398	(92.99%)	30	(7.01%)	(4.78–9.86)	
***Health characteristics*** ^g^						
Prior or present symptoms of SARS-CoV-2 disease						
No	365	(90.12%)	40	(9.88%)	(7.15–13.21)	**<0.001**
Yes	23	(53.49%)	20	(46.51%)	(31.18–62.35)	
Tobacco consumption						
No	424	(91.58%)	39	(8.42%)	(6.06–11.34)	**<0.001**
Yes	590	(97.04%)	18	(2.96%)	(1.76–4.64)	
Alcohol consumption						
No	717	(93.6%)	49	(6.40%)	(4.77–8.37)	0.047
Yes	277	(96.85%)	9	(3.15%)	(1.45–5.89)	
Comorbidity						
No	513	(95.18%)	26	(4.82%)	(3.17–6.99)	0.307
Yes	578	(93.68%)	39	(6.32%)	(4.53–8.54)	
Number of comorbidities	1	(1.3)	1	(1.1)		0.749
Psychiatric and addiction comorbidities						
No	827	(93.55%)	57	(6.45%)	(4.92–8.27)	**0.034**
Yes	264	(97.06%)	8	(2.94%)	(1.28–5.71)	
Risk factors for severe SARS-CoV-2 disease						
Obesity						
No	1000	(94.61%)	57	(5.39%)	(4.11–6.93)	0.594
Yes	69	(93.24%)	5	(6.76%)	(2.23–15.07)	
Diabetes						
No	1012	(94.4%)	60	(5.6%)	(4.3–7.15)	0.807
Yes	79	(94.05%)	5	(5.95%)	(1.96–13.35)	
Cancer						
No	1045	(94.74%)	58	(5.26%)	(4.02–6.74)	0.124
Yes	20	(86.96%)	3	(13.04%)	(2.78–33.59)	
Chronic Respiratory Disease						
No	935	(94.35%)	56	(5.65%)	(4.3–7.28)	0.812
Yes	88	(95.65%)	4	(4.35%)	(1.2–10.76)	
Cardiovascular Disease						
No	884	(94.24%)	54	(5.76%)	(4.35–7.45)	0.847
Yes	140	(95.24%)	7	(4.76%)	(1.94–9.57)	
Chronic Kidney Disease						
No	1001	(94.34%)	60	(5.66%)	(4.34–7.22)	0.999
Yes	21	(95.45%)	1	(4.55%)	(0.12–22.84)	

95%IC: 95% confidence interval; SD: standard deviation.

*ETHOS: the European typology for homelessness and housing exclusion.

^§^: “European Union” countries: Belgium, Bulgaria, Germany, Hungary, Italy, Poland, Portugal, Romania, Czech Republic, Slovakia, and Spain. “Non-EU European” countries: Albania, Armenia, Bosnia, Croatia, Moldavia, Montenegro, Serbia, Russia including Chechenia and Ukraine.

Seroprevalence was lower in participants who reported tobacco consumption (2.96% [1.76–4.64]) than in non-tobacco users (8.42% [6.06–11.34]) (P < 0.001) (Table 3). Seroprevalence was 2.2-fold lower in participants with psychiatric and/or addiction comorbidities (2.94% [(1.28–5.71]) compared to their counterparts without psychiatric and/or addiction comorbidities (6.45% [4.92–8.27]) (P = 0.034). Almost half of the participants who had symptoms at the time of testing were SARS-CoV-2 seropositive compared to 6% among participants who reported no symptoms (P < 0.001).

## Discussion

This study is the first attempt to quantify the seroprevalence of SARS-CoV-2 among homeless people using a systematic approach taking into account different types of accommodations.

The primary key finding of this study is that emergency shelters represented the greatest risk of SARS-CoV-2 exposure. With an estimated 8%, the seroprevalence among homeless people living in emergency shelters was twofold higher than the national seroprevalence survey (EPICOV) [21]. This French seroprevalence study based on 12,000 national samples collected between May and June, 2020 reported a positivity rate of 4.5% throughout French territory and a positivity rate of 3.6% specifically in Marseille. The results from our survey match attempts to quantify over-exposure to SARS-CoV-2 in homeless people throughout other countries. Tobolowsky and colleagues reported prevalence data from three affiliated homeless shelters in Seattle, Washington, during the period March 30 –April 11, 2020. Among the 245 residents tested using a SARS-CoV-2 PCR assay with a nasopharyngeal swab, 18% had positive test results [14]. Baggett and colleagues reported results for SARS-CoV-2 infection prevalence in residents of a large homeless shelter in Boston. Between March and April 2020, 408 residents were tested, and 36% of them were positive for a SARS-CoV-2 PCR test [12]. Both studies recruited homeless shelters with a COVID-19 case cluster, which is clearly different from our systematic approach. Similarly, SARS-CoV-2 seroprevalence studies from cohorts representing the general population or healthcare personnel have reported heterogeneous estimates from April to early May this year [22–27]. These mainly nationwide population-based cohort studies found seroprevalence rates ranging from 1.0% in the San Francisco Bay area in April 2020, to 8.5% in the canton of Geneva in May 2020. However, as previously mentioned, these seroprevalence data collections were spread over a significantly different time period, which makes comparable figures hard to find.

While our results suggest that, lengths of stay in emergency shelters for homeless individuals should be as short as possible to minimize morbidity related to SARS-CoV-2, rapid access to affordable housing and support is too often missing and homeless people are still rotating between streets, shelters, squats, and hospital [28, 29]. If resources are not provided for housing, less overcrowded accommodations with a higher proportion of social workers like transitional shelters would appear to be more appropriate and safer to prevent exposure to a pandemic.

Our overall seroprevalence estimate in the study population of people experiencing homelessness was 5.6% compared to 3.6% in the general population in Marseille. At the same period, the prevalence for SARS-CoV-2 based on molecular testing was 1.3% on the French territory and 1.8% in the South-East region where Marseille is located [30]. In Marseille, existing official data on COVID-19 showed that the first case in the general population was diagnosed on March 3, 2020 and the epidemic remained active until the end of the study period, with an incidence rate > 70/100,000 inhabitants. Although our homeless population experienced an increased risk of SARS-CoV-2 infection compared to the general population, this population appears to have remained relatively unexposed to SARS-CoV-2, even in an area with widespread virus circulation. Public health measures such as making tourist hotel rooms available, reducing population density in emergency shelters, and testing campaigns including this study, probably contributed to preventing the spread of SARS-CoV-2 within the homeless population.

Since social contacts are the means of propagation of SARS-CoV-2 infection, lower seroprevalence among homeless people living rough and among homeless people with psychiatric disorders should be interpreted as a sign that these particularly stigmatized people are excluded [31]. Qualitative research was performed in conjunction with the present epidemiology study [32], which revealed insights into how homeless people have strong individual and group health skills, but the availability of resources like water or safe, affordable housing prevented people from being able to protect themselves from the SARS-CoV-2 pandemic.

Our study led us to question the screening tests we used for this large homeless population. Methods of screening have recently made enormous progress with the availability of rapid tests that can be used at a community level [33, 34]. As noted by WHO, “rapid serology tests, applied in the right situation for appropriate public health measures to be put into place, can make a huge difference” [35]. For SARS-CoV-2, rapid antibody and molecular tests were available from March 2020, with good to high sensitivity and specificity [36–39]. Detection of antibodies to SARS-CoV-2 takes a different approach to existing virological diagnosis approaches, aiming to assess the exposure of a broader population to a virus and to indicate that people had been infected at some point since the start of the pandemic. For example, a large majority of our seropositive population did not report any prior or recent symptoms compatible with SARS-CoV-2 infection. This suggests that rapid serologic assays represent appropriate tools for homelessness services to help them discriminate against infection and set up more effective public health measures in the homeless population, who are accustomed to living with symptoms associated with chronic diseases, and therefore possibly underestimate the symptoms of SARS-CoV-2 infection.

This study is a cross sectional study conducted during the epidemic outbreak in order to inform public decision-makers of the health and social concerns of the homeless population in Marseille, and to discuss the disparities observed in exposure to COVID-19 according to their type of accommodations. The most immediate and evident conclusion that can be drawn from this study is that the COVID-19 pandemic affected the population under survey more than the French general population. These results contribute to highlight the need to organize regular screening to prevent (rather than trace) clusters in homeless accommodations and to maintain specific housing solutions for the homeless during the pandemic. These solutions must include re-housing, a ban on squat evictions and less populated settings with adequate prevention measures.

## Supporting information

S1 File COVID_Homeless survey. English Version and French Version.
(PDF)
