# Supplementary material for: Seroprevalence of SARS-CoV-2 antibodies among homeless people living rough, in shelters and squats: A large population-based study in France
Source: PLoS One. 2021 Sep 15;16(9):e0255498. doi: 10.1371/journal.pone.0255498 (PMC8443066; doi:10.1371/journal.pone.0255498)
Supplement: S1 File — English Version and French Version. (PDF) [file pone.0255498.s001.pdf]

ENGLISH VERSION

SEROPREVALENCE STUDY COVID_HOMELESS SURVEY

Inclusion number ___________________________________
First letter of last name ___________________________________
First letter of first name ___________________________________
If living in the street (ETHOS1), give the usual living place ____________________________

Year of birth or estimated age ___________________________________
Inclusion date ___________________________________

General Data

Social and administrative data

Year of birth ___________________________________
Or Estimated age ___________________________________
Gender (at birth) man / woman / intergender
Gender (chosen) male / female / intergender
Mode de vie: single adult / adult couple/family / single adult in extended family / single parent
Number of children living with the person (dependent children) ________________________________
Children followed by a health professional yes (family doctor, paediatrician, at a hospital, PMI) / only those aged 6 to 15 years? / no / other
If other, state what: ___________________________________
Do the children go to school? Yes / No
Country where the person was born:
(North Africa: Algeria, Morocco, Tunisia, Egypt, Libya, West Sahara) / France / EU country / Non-EU European country / North African country / Sub-Saharan or South African country / Middle Eastern country / Russia or non-EU country close to Russia / North America / South America / Other
Nationality clearly stated:___________________________________
Town where the person was born: ___________________________________
(Only for people born in France)
The person does not speak French ("No" means French-speaking): Yes / No
If Yes, which languages do they speak: English / Arab / Albanian / German / Spanish / Italian / Kurdish / Kurmanji / Pashto / Polish / Portuguese / Romanian / Russian /Swahili / Other
If Other, which one(s): ___________________________________
If Yes, what interpreting service is needed? None / family or friend / professional interpreter by phone / health mediator/other (including other professional)

Housing

Main living place before lockdown (16 March 2020): Street / Emergency community shelter / Hotel financed by emergency housing / Hotel personally financed / Temporarily lodged by family (friends) / Squat in a lodging: no (sub)rental contract / Slum: land or building occupation, not accommodation / CHRS / ACT / LHSS / Hospital / Personal apartment / Other
If Other, state where: ___________________________________
Main living place during lockdown (16 March – 11 May): Street / Emergency community shelter / Hotel financed by emergency housing / Hotel personally financed / Temporarily lodged by family (friends) / Squat in a lodging: no (sub)rental contract / Slum: land or building occupation, not accommodation / CHS Covid 19 / CHRS / ACT / LHSS / Hospital / Personal apartment / Other
If Other, state where: ___________________________________
Main living place after lockdown (11 May): Street / Emergency community shelter / Hotel financed by emergency housing / Hotel personally financed / Temporarily lodged by family (friends) / Squat in a lodging: no (sub)rental contract / Slum: land or building occupation, not accommodation / CHS Covid 19 / CHRS / ACT / LHSS / Hospital / Personal apartment / Other
If Other, state where: ___________________________________

Number of people in close contact with the person during the day and night (today)_______________
(close contact = less than one metre away for over 15 minutes)
Length of time spent homeless throughout whole life: Less than 3 months / Less than 1 year / From 1 to 5 years / Over 5 years

Socio-economic data

What were the person’s sources of income before this health crisis? Work / Undeclared work / Unemployment benefits / RSA / Asylum seeker benefits / Family allowance / AAH / Retirement pension / Disability pension / Financial help from friend/family / Begging / Does not know or does not want to answer / None / Other
If Other, state what: ___________________________________
What are the person’s sources of income now?
Work / Undeclared work / Unemployment benefits / RSA / Asylum seeker benefits / Family allowance / AAH / Retirement pension / Disability pension / Financial help from friend/family / Begging / Does not know or does not want to answer / None / Other
If Other, state what: ___________________________________
Have there been any economic issues during this health crisis? Yes / No
What is the person’s level of education? No diploma / CAP or BEP or apprentice certificate or BEPC or Brevet collège / Bac or equivalent / 1st cycle University or DUT or BTS / >=2nd cycle University or equivalent / Don’t know

Medical Record

Usual socio-health follow-up and upon inclusion

What health insurance coverage? (if in difficulty, offer options)
If no cover, are there pending procedures underway? Yes / No
Usual health worker (name, facility, contact) ___________________________________
Usual social worker (name, facility, contact) ___________________________________
The person is aged 50 years or over: Yes / No
The person is pregnant (>= second term): Yes / No
The person is obese (BMI > 30 kg/m²): Yes / No
Diabetes: Yes / No
Cancer under therapy: Yes / No
Psychiatric or addiction issues: Yes / No
Decompensation of one (several) chronic disease(s) since the beginning of lockdown? Yes / No
If Yes, which one(s)? Cardiac / Psychiatric / Respiratory / HIV, HCV, HBV / Endocrine / Other
Has the person already caught SARS-CoV-2? Yes / No / Don’t know

COVID-19 previous history

Has the person had clinical signs that are compatible with COVID-19 since the beginning of the pandemic (end February 2020)? Yes / No / Don’t know
(fever and/or flu symptoms, cough, difficulty breathing, headache, diarrhoea, confusion, loss of smell/taste etc.)
Has the person been admitted to hospital for COVID-19 (or suspected) since the beginning of the pandemic? Yes / No
If Yes, dates of hospitalization ___________________________________
If Yes, place of hospitalization ___________________________________

Previous medical and comorbidity history

(filled in using the person’s medical record)

History of Cardiovascular pathologies:
(Hypertension complications, stroke or coronary disease, heart surgery, heart failure NYHA class III or IV): Yes / No
Chronic respiratory disease susceptible to decompensation upon viral infection (COPD class III or IV, chronic pulmonary disease, asthma under dual-therapy, cystic fibrosis): Yes / No
Cirrhosis ≥ class B of Child-Pugh classification: Yes / No
Chronic kidney disease under dialysis: Yes / No
Malignant blood disease under therapy: Yes / No
Congenital or acquired immunodeficiency: Yes / No
Post-transplant immunodeficiency (organ or stem-cells): Yes / No

Medication-induced immunodeficiency (cancer chemotherapy, immunosuppression, biotherapy and/or immunosuppressive cortisone therapy): Yes / No
Uncontrolled HIV or T-CD4< 200/mm3: Yes / No
Homozygous sickle-cell disease or splenectomy: Yes / No
History of lung tuberculosis or latent tuberculosis: Yes / No

Long-term treatments

Long-term usual treatment: Yes / No
If Yes, state what:
NSAID
ACE inhobitors / ARB
Hypnotics
Antidepressants
Neuroleptics
Anxiolytics
Mood stabilizers
Addiction substitution therapy (OST)
Other analgesics
Long-term cortisone therapy
Other
If Yes, state clearly the treatments, with dosage
Treatment 1 ___________________________________
Treatment 2 ___________________________________
Treatment 3 ___________________________________

Vaccination

Flu vaccine (this year): Yes / No / Don't know
BCG vaccine: Yes / No / Don't know

The person's needs (since the beginning of the health crisis)

Difficulties connected to health

Since the beginning of the health crisis, the person has needs connected to his/her state of health (somatic illnesses)?: Yes / No
If Yes, state what: ___________________________________
Since the beginning of the health crisis, the person has needs connected to mental/psychiatric issues (melancholy, anxiety, agitation, bizarrerie, delirium etc.): Yes / No
If Yes, state what: ___________________________________
Since the beginning of the health crisis, the person has needs connected to addiction (product use, withdrawal, etc.): Yes / No
If Yes, state what: ___________________________________
Since the beginning of the health crisis, the person has needs connected to a lack of autonomy (disability): Yes / No
If Yes, state what: ___________________________________

Basic needs/ Housing

Since the beginning of the health crisis, the person has had difficulty gaining access to
FOOD? never/rarely /sometimes /often / always
If Yes, say what: ___________________________________
RUNNING WATER? never/rarely /sometimes /often / always
If Yes, say what: ___________________________________
HYGIENE PRODUCTS? never/rarely /sometimes /often / always
If Yes, say what: ___________________________________
CLEANING PRODUCTS? never/rarely /sometimes /often / always
If Yes, say what: ___________________________________
HOUSING COMPATIBLE WITH LOCKDOWN? never/rarely /sometimes /often / always
If Yes, say what: ___________________________________
SOCIAL WORKER ASSISTANCE? never/rarely /sometimes /often / always
If Yes, say what: ___________________________________

Needs / resources connected to Entourage
Is there anyone vulnerable in the person's close entourage? (pregnant woman, old person, person with chronic illness) (since the beginning of the health crisis): Yes / No
Can the person count on anyone in his/her entourage? (since the beginning of the health crisis): Yes / No

Consumption/ Addiction

Tobacco smoker: Yes / No
If Yes, estimate the number of Packets per Annum (ex: 1 packet/day for 10 years = 10 PA, 2 packets per day for 10 years = 20 PA) : ___________________________________
Alcohol consumption: Yes / No
If Yes, estimate the number of glasses drunk/day (1 standard glass = 1 glass of red wine = 25cl of beer = 1 shot of spirit = 1 Ricard etc. Be careful, glasses served among friends, or strong beers do not correspond to these standard glasses) : < 3 glasses/day standard / 3 glasses/day / > 3 glasses/day
Current consumption of toxic psychoactive drugs/other drugs: Yes / No
If Yes, state what:
(Past or present) drug injecting behaviour? Yes / No
Drug injection within the last 6 months? Yes / No

Suspicion of Covid19: Diagnostic assays

Serological assays Ab - M0

Was the consent form signed? Yes / No
If Yes, date of consent: ___________________________________
Was the serological assay done? Yes / No
If Yes, which assay was used?
Rapid serological assay (TROD), fingertip
Classical serological assay (ELISA)
Seroneutralization assay (blood taken by the research team nurse)
If Yes, date of serological assay : ___________________________________
If not tested, why not? Sample not asked for / Sample not given / Consent form not signed / Other
If Other, state what: ___________________________________
Date of TROD : ___________________________________
TROD results: Presence IgM: Yes / No
TROD results: Presence IgG: Yes / No

Other serological results (research team)

Classical serological assay (ELISA)results: Presence of IgM / Presence of IgG / Non contributive results / IgM not tested for / IgG not tested for
Seroneutralization assay results: Presence of antibodies in serum? Yes / No
If Yes, titer (1/XX): ___________________________________

Clinical signs

Were there any clinical signs of Covid19? Yes / No
If Yes, what were the clinical signs?
Fever > 38°C
Febrile state with no fever
Cough
Dyspnea
Headache
Anosmia/Ageusia (loss of smell/taste)
Rhinitis
Asthenia/Fatigue
Diarrhoea
Arthromyalgia (aching joints and muscles)
Odynophagia (pain swallowing)
Shivering
Marbling
Skin rash

Conjunctivitis
Other
If Yes, when? (approximate date when signs began) ___________________________________
If Yes, when? (approximate date when signs ended) ___________________________________
If Yes, where? (where was the person living when the person experienced the symptoms?)
Street (alone)
Street (with other people)
Emergency accommodation (private bedroom)
Emergency accommodation (bedroom shared with other people)
Hotel (private bedroom)
Hotel (bedroom shared with other people)
Squat/slum (private bedroom)
Squat/slum (bedroom shared with other people)
Hospital
Lodged by family/friends (private bedroom)
Lodged by family/friends (bedroom shared with other people)
Other facilities (private bedroom)
Other facilities (bedroom shared with other people)
Private apartment

Diagnostic assays: PCR, POC

Date of Covid19 PCR test (proposed in first line) __________________________________
Covid19 PCR test results: Positive / Negative / Unknown
Date of rapid Covid19 virological assay – POC: ____________________
Rapid Covid19 virological assay – POC: Positive / Negative / Unknown

Housing conditions since the beginning of the health crisis

What are the person’s current housing conditions?

Street (alone)
Street (with other people)
Emergency accommodation (private bedroom)
Emergency accommodation (bedroom shared with other people)
Hotel (private bedroom)
Hotel (bedroom shared with other people)
Squat/slum (private bedroom)
Squat/slum (bedroom shared with other people)
Hospital
Lodged by family/friends (private bedroom)
Lodged by family/friends (bedroom shared with other people)
Other facilities (private bedroom)
Other facilities (bedroom shared with other people)
Private apartment
(Considered to be a private bedroom if the couple or family are in the same bedroom)
How many nights has the person been sleeping in this place? (number of nights)______________________
Since the beginning of the health crisis has the person changed accommodation at least once? Yes / No

Before living in the current accommodation/place, where was the person living?

Street (alone)
Street (with other people)
Emergency accommodation (private bedroom)
Emergency accommodation (bedroom shared with other people)
Hotel (private bedroom)
Hotel (bedroom shared with other people)
Squat/slum (private bedroom)
Squat/slum (bedroom shared with other people)
Hospital
Lodged by family/friends (private bedroom)
Lodged by family/friends (bedroom shared with other people)

Other facilities (private bedroom)
Other facilities (bedroom shared with other people)
Private apartment
For approximately how many nights?:________________________________

And before then?

Street (alone)
Street (with other people)
Emergency accommodation (private bedroom)
Emergency accommodation (bedroom shared with other people)
Hotel (private bedroom)
Hotel (bedroom shared with other people)
Squat/slum (private bedroom)
Squat/slum (bedroom shared with other people)
Hospital
Lodged by family/friends (private bedroom)
Lodged by family/friends (bedroom shared with other people)
Other facilities (private bedroom)
Other facilities (bedroom shared with other people)
Private apartment
For approximately how many nights?:________________________________

Following COVID-19 recommendations

Respecting lockdown (during the lockdown period, from 16 March to 11 May)

Was the person able to have a private bedroom (or as a couple or family) during lockdown?: Yes / No
If No, why not? No private bedroom offered or available / Refused by the person / Other
Was lockdown respected (restricted outings, less than 1km, contacts < 5/day)? rather yes / rather no
Did the person understand lockdown? rather yes / rather no

Respecting protective measures (during the lockdown period, from 16 March to 11 May)

Application of hand washing several times a day and/or hand sanitizers? rather yes / rather no
If rather no, why not? No soap / No hand sanitizer / Difficult access to water / Difficulty understanding / Does not feel concerned
Application of social distancing > 1 metre? rather yes / rather no
If rather no, why not? Difficulty understanding / Does not feel concerned / Distancing impossible (community settings) / Distancing culturally inconceivable / Other
Application of mask wearing? rather yes / rather no
If rather no, why not? Masks not provided / Masks not tolerated / Difficulty understanding / Refusal, does not feel concerned

VERSION FRANCAISE

QUESTIONNAIRE ETUDE SEROPREVALENCE COVID

Numéro d'inclusion ___________________________________
Première lettre du nom ___________________________________
Première lettre du prénom ___________________________________
Si personne vivant à la rue (ETHOS1), indiquer lieu de vie habituel ____________________________

Année de naissance ou âge évalué ___________________________________
Date d'inclusion ___________________________________

Données Générales

Données sociales et administratives

Année de naissance ___________________________________
Ou Age évalué ___________________________________
Sexe (de naissance) homme / femme / intersexe
Genre (choisi) masculin / féminin / intergenre
Mode de vie : adulte isolé / adulte en couple/famille / adulte célibataire en famille élargie / parent isolé
Nombre d'enfants vivant avec la personne (enfants à charge) ________________________________
Enfant(s) suivi(s) par un professionnel de santé oui (médecin de famille, pédiatre, à l'hôpital, PMI) / que ceux de 6 à 15 ans ? / non / autre
Si autre, précisez : ___________________________________
Les enfants vont-ils à l'école? Oui /Non
Pays de naissance de la personne :
(Afrique du nord : Algérie, Maroc, Tunisie, Egypte, Libye, Sahara Occ) / France / Pays européen de l'UE / Pays européen hors UE / Pays d'Afrique du Nord / Pays d'Afrique subsaharienne ou australe / Pays du proche Orient / Russie ou pays en périphérie de la Russie hors UE / Amérique du nord / Amérique du sud / Autre
Nationalité en clair :___________________________________
Ville de naissance de la personne : ___________________________________
(Seulement pour les personnes nées en France)
La personne est-elle allophone? (CAT: ne parle pas français. "Non" signifie francophone) : Oui / Non
("Non" signifie francophone)
Si allophone, langues pratiquées : Anglais / Arabe / Albanais / Allemand / Espagnol / Italien / Kurde / Kurmandji / Pachto / Polonais / Portugais / Roumain / Russe /Swahili / Autre
Si autre, la(les)quelle(s) : ___________________________________
Si oui, quels sont les besoins en interprétariat? Aucun /entourage/ interprétariat professionnel téléphonique /médiateur en santé / autre (dont autre professionnel)

Hébergement

Lieu de vie principal avant le début du confinement (16 mars) : Rue / Hébergement d'urgence collectif / Hôtel financé par l'hébergement d'urgence / Hôtel en autofinancement / Hébergé(e) provisoirement dans la famille (chez des amis) / Squat d'un logement : sans bail de (sous)location / Bidonville : occupation de terrain ou bâtiment, hors logement / CHRS / ACT / LHSS / Hôpital / Appartement personnel / Autres
Si autres, précisez ___________________________________
Lieu de vie principal de la personne pendant le confinement (16 mars - 11 mai) : Rue / Hébergement d'urgence collectif / Hôtel financé par l'hébergement d'urgence / Hôtel en autofinancement / Hébergé(e) provisoirement dans la famille (chez des amis) / Squat d'un logement : sans bail de (sous)location / Bidonville : occupation de terrain ou bâtiment, hors logement / CHS Covid19/ CHRS / ACT / LHSS / Hôpital / Appartement personnel / Autres
Si autres, précisez : ___________________________________
Lieu de vie principal de la personne après la fin du confinement (le 11 mai) : Rue / Hébergement d'urgence collectif / Hôtel financé par l'hébergement d'urgence / Hôtel en autofinancement / Hébergé(e) provisoirement dans la famille (chez des amis) / Squat d'un logement : sans bail de (sous)location / Bidonville : occupation de terrain ou bâtiment, hors logement / CHS Covid19/ CHRS / ACT / LHSS / Hôpital / Appartement personnel / Autres

Si autres, précisez : ___________________________________
Nombre de personnes en contact rapproché pendant la journée et la nuit (ce jour) _______________
(contact rapproché = moins d'un mètre plus de 15 minutes)
Durée de vie sans chez-soi cumulé sur la vie entière : Moins de 3 mois / Moins d'1 an / De 1 à 5 ans / Plus de 5 ans

Données socio-économiques

Quelles étaient les sources de revenus avant la crise sanitaire? Travail / Travail non déclaré / Alloc chômage / RSA / Alloc demandeurs asile / Alloc familiale / AAH /Retraite / Pension invalidité / Aide financière ami/famille / Mendicité / Ne sait pas ou ne veut pas répondre / Aucune / Autres
Si autre, précisez : ___________________________________
Quelles sont les sources de revenus de la personne aujourd'hui?
Travail / Travail non déclaré / Alloc chômage / RSA / Alloc demandeurs asile / Alloc familiale / AAH /Retraite / Pension invalidité / Aide financière ami/famille / Mendicité / Ne sait pas ou ne veut pas répondre / Aucune / Autres
Si autre, précisez : ___________________________________
Y a t il eu des problèmes de ressources économiques pendant la période de crise sanitaire? Oui / Non
Quel est le niveau d'études de la personne? Sans diplôme / CAP ou BEP ou certificat apprentissage ou BEPC ou Brevet collège / Bac ou équivalent / 1er cycle universitaire ou DUT ou BTS / >=2e cycle universitaire ou équivalent / Ne sait pas

Dossier Médical

Suivi socio-sanitaire habituel et à l'inclusion

Quelle couverture sociale ? (si difficulté, citer les possibilités)
Si aucune couverture, y a-t-il des démarches déjà en cours et en attente de réponse ? Oui / Non
Référent sanitaire habituel (nom, structure, contact) ___________________________________
Référent social habituel (nom, structure, contact) ___________________________________
La personne est âgée de 50 ans ou plus : Oui/ Non
La personne est une femme enceinte (dès le second trimestre) : Oui / Non
La personne est obèse (IMC > 30 kg/m²) : Oui / Non
Diabète : Oui / Non
Cancer sous traitement : Oui / Non
Des troubles psychiatriques ou des addictions : Oui / Non
Décompensation d'une (plusieurs) pathologie(s) chronique(s) depuis début du confinement ? Oui / Non
Si oui, laquelle/lesquelles? Cardiaque / psychiatrique / pneumologique / VIH, VHC, VHB / endocrinologie / autre
Est-ce que la personne a déjà contracté le virus SARS-CoV-2? Oui / Non / Ne sait pas

Antécédents de COVID-19

Est-ce que la personne a présenté des signes cliniques compatibles avec le COVID-19 depuis le début de la pandémie (fin février 2020)? Oui / Non / Ne sait pas
(fièvre et/ou sd grippal, toux, difficultés respiratoires, céphalées, mal de gorge, diarrhée, confusion, perte odorat/goût,..)
Est-ce que la personne e été hospitalisée pour COVID-19 (ou suspicion) depuis le début de la pandémie? Oui / Non
Si oui, date d'hospitalisation ___________________________________
Si oui, lieu d'hospitalisation ___________________________________

Antécédents médicaux et comorbidités

(à renseigner à l'aide du dossier médical de la personne)

Antécédents/Pathologies cardiovasculaires :
HTA compliquée, AVC ou coronaropathie, chir. Cardiaque, IC stade NYHA III ou IV) : Oui / Non
Pathologie chronique respiratoire susceptible de décompenser lors d'une infection virale (BPCO stade III ou IV, insuffisance respiratoire chronique, asthme sous bithérapie, mucoviscidose) : Oui / Non
Cirrhose ≥ stade B de la classification de Child-Pugh : Oui/ Non
Insuffisance rénale chronique dialysée Oui / Non
Hémopathie maligne sous traitement Oui / Non
Immunodépression congénitale ou acquise Oui / Non
Immunodépression post-greffe (organe ou cell souches) Oui / Non

Immunodépression médicamenteuse (chimiothérapie anti-cancéreuse, immunosuppresseur, biothérapie et/ou corticothérapie à dose immunosuppressive) : Oui / Non
VIH non contrôlée ou avec CD4< 200/mm3 Oui / Non
Drépanocytose homozygote ou splénectomie Oui / Non
Antécédents de Tuberculose pulmonaire ou tuberculose latente : Oui / Non

Traitements au long cours

Traitement habituels au long cours Oui / Non
Si oui, précisez :
AINS
IEC / ARA
Hypnotiques
Antidépresseurs
Neuroleptiques
Anxiolytiques
Thymo-régulateurs
Traitement substitutif d'une addiction (TSO)
Antalgiques autres
Corticothérapie au long cours
Autres
Si oui, traitements habituels en clair, avec les posologies
Traitement 1 ___________________________________
Traitement 2 ___________________________________
Traitement 3 ___________________________________

Vaccins

Vaccination anti-grippe (dans l'année) Oui / Non / Ne sait pas
Vaccin BCG Oui / Non / Ne sait pas

Besoins de la personne (depuis le début de la crise sanitaire)

Difficultés liées à l'état de santé

Depuis le début de la crise sanitaire, la personne a des besoins liés à son état de santé (maladies somatiques) ? (depuis le début de la crise sanitaire) : Oui / Non
Si oui, précisez : ___________________________________
Depuis le début de la crise sanitaire, la personne a des besoins liés à des problèmes psychique/psychiatrique (tristesse, anxiété, agitation, bizarrerie, délire...) (depuis le début de la crise sanitaire) : Oui / Non
Si oui, précisez ___________________________________
Depuis le début de la crise sanitaire, la personne a des besoins liés des addictions (prise de produits, en manque, etc.) (depuis le début de la crise sanitaire) : Oui / Non
Si oui, précisez : ___________________________________
Depuis le début de la crise sanitaire, la personne a des besoins liés à un déficit d'autonomie (handicap) ? (depuis le début de la crise sanitaire) : Oui / Non
Si oui, précisez : ___________________________________

Besoins primaires/ Hébergement

Depuis le début de la crise sanitaire, la personne a eu des difficultés à avoir accès à la
NOURRITURE? Jamais /rarement /parfois / souvent /toujours
Si oui, précisez : ___________________________________
EAU COURANTE? Jamais /rarement /parfois / souvent /toujours
Si oui, précisez : ___________________________________
PRODUITS D HYGIENE? Jamais /rarement /parfois / souvent /toujours
Si oui, précisez : ___________________________________
PRODUITS DE NETTOYAGE? Jamais /rarement /parfois / souvent /toujours
Si oui, précisez : ___________________________________
HEBERGEMENT COMPATIBLE AVEC UN CONFINEMENT? Jamais /rarement /parfois / souvent /toujours
Si oui, précisez : ___________________________________
AIDE D UN TRAVAILLEUR SOCIAL? Jamais /rarement /parfois / souvent /toujours
Si oui, précisez : ___________________________________

Besoins / ressources liés à l'Entourage
Dans l'entourage proche, y a-t-il une ou des personnes fragiles? (femmes enceinte, personne âgée, personne avec maladie chronique) (depuis le début de la crise sanitaire) : Oui / Non
Est-ce que la personne peut compter sur quelqu'un dans son entourage? (depuis le début de la crise sanitaire) : Oui / Non

Consommations/ Addictions
Tabagisme actif :Oui / Non
Si oui, estimer la consommation vie entière en Paquets Années (ex: 1 paquet/jour pendant 10 ans = 10 PA, 2 paquets par jours pdt 10 ans = 20 PA) : ________________________________
Consommation d’Alcool : Oui / Non
Si oui, estimer la consommation en nombre de verre " /jour (1 verre standard = 1 ballon de rouge = 1 demi de bière = 1 shoot d'alcool fort = 1 ricard etc. Attention les quantités servies entre amis, ou les bières fortes ne correspondent pas à ces verres standards.) : < 3 verres/j standard / 3 verres/j / > 3 verres/j
Prise actuelle de substances psychoactives toxiques / autres drogues : Oui / Non
Si oui, précisez :
Comportement (actuel ou dans le passé) d'injection de substances? Oui / Non
Injection de substances au cours des 6 derniers mois? Oui / Non

Suspicion Covid19 : Tests diagnostiques
Tests Sérologiques Ac - M0
Le consentement a-t-il été signé? Oui/ Non
Si oui, date consentement : ________________________________
Le test sérologique a-t-il été réalisé? Oui / Non
Si oui, quel test sérologique est réalisé?
TROD (test rapide d'orientation diagnostique), bout du doigt
Sérologie classique (ELISA)
Immuno-séroneutralisation (prise de sang avec IDE de l'équipe de recherche)
Si oui, date de réalisation test sérologique : ________________________________
Si test non réalisé, pourquoi? prélèvement non proposé / prélèvement non accepté / consentement non signé / autre
Si autre, précisez : ________________________________
Date TROD : ________________________________
Résultat TROD : Présence IgM : Oui / Non
Résultat TROD : Présence IgG : Oui / Non

Résultats autres sérologies (équipe recherche)
Résultats Sérologie classique (ELISA) :Présence d'IgM / Présence d'IgG / Résultats non contributifs / IgM non recherchés /IgG non recherchés
Résultats Immunoséreoneutralisation : Présence d'ac séroneutralisant ? Oui / Non
Si oui, titre (1/XX) : ________________________________

Signes cliniques
Y a t il eu des signes cliniques de Covid19? Oui / Non
Si oui, quels ont été les signes cliniques?
Fièvre > 38°C
Sd fébrile sans fièvre
Toux
Dyspnée
Céphalées
Anosmie/Agueusie (perte odorat/goût)
Rhinite
Asthénie/Fatigue
Diarrhées
Arthro-myalgies (courbatures)
Odynophagie (mal de gorge)
Frissons
Marbrures

Eruption cutanée
Conjonctivite
Autre
Si oui, quand? (date approximative de début des signes) ___________________________________
Si oui, quand? (date approximative de fin des signes) ___________________________________
Si oui, où? (où logeait/était la personne lorsque elle a ressenti les symptômes?)
Rue (seul/e)
Rue (avec d'autres personnes)
Hébergement d'urgence (chambre individuelle)
Hébergement d'urgence (chambre partagée avec d'autres personnes)
Hôtel (chambre individuelle)
Hôtel (chambre partagée avec d'autres personnes)
Squat/bidonville (chambre individuelle)
Squat/bidonville (chambre partagée avec d'autres)
Hôpital
Hébergé(e) dans la famille/chez des amis (chambre individuelle)
Hébergé(e) dans la famille/chez des amis (chambre partagee)
Autre structures (chambre individuelle)
Autre structures (chambre partagée)
Appartement personnel

Tests diagnostiques : PCR, POC

Date de Test Covid19 par PCR (proposé en 1ère intention) ___________________________________
Résultat de test Covid19 par PCR : Positif / Négatif / Non connu
Date de Test virologique Covid19 rapide - POC : ____________________
Résultat de test virologique Covid19 rapide -POC : Positif / Négatif / Non connu

Parcours d'hébergement depuis le début de la crise sanitaire

Quel est le logement actuel de la personne?

Rue (seul/e)
Rue (avec d'autres personnes)
Hébergement d'urgence (chambre individuelle)
Hébergement d'urgence (chambre partagée avec d'autres personnes)
Hôtel (chambre individuelle)
Hôtel (chambre partagée avec d'autres personnes)
Squat/bidonville (chambre individuelle)
Squat/bidonville (chambre partagée avec d'autres)
Hôpital
Hébergé(e) dans la famille/chez des amis (chambre individuelle)
Hébergé(e) dans la famille/chez des amis (chambre partagée)
Autre structures (chambre individuelle)
Autre structures (chambre partagée)
Appartement personnel
(Considérer comme chambre individuelle si chambre en couple ou en famille)
Depuis combien de nuits la personne vit à cet endroit? (nombre de nuits) _____________________
Depuis le début de la crise sanitaire, est-ce que la personne a changé d'hébergement au moins une fois? Oui / Non

Avant d'occuper le logement/endroit actuel, où vivait la personne?

Rue (seul/e)
Rue (avec d'autres personnes)
Hébergement d'urgence (chambre individuelle)
Hébergement d'urgence (chambre partagée avec d'autres personnes)
Hôtel (chambre individuelle)
Hôtel (chambre partagée avec d'autres personnes)
Squat/bidonville (chambre individuelle)
Squat/bidonville (chambre partagée avec d'autres)
Hôpital
Hébergé(e) dans la famille/chez des amis (chambre individuelle)

Hébergé(e) dans la famille/chez des amis (chambre partagée)
Autre structures (chambre individuelle)
Autre structures (chambre partagée)
Appartement personnel
Pour combien de nuits environ? :________________________________

Et encore avant?

Rue (seul/e)
Rue (avec d'autres personnes)
Hébergement d'urgence (chambre individuelle)
Hébergement d'urgence (chambre partagée avec d'autres personnes)
Hôtel (chambre individuelle)
Hôtel (chambre partagée avec d'autres personnes)
Squat/bidonville (chambre individuelle)
Squat/bidonville (chambre partagée avec d'autres)
Hôpital
Hébergé(e) dans la famille/chez des amis (chambre individuelle)
Hébergé(e) dans la famille/chez des amis (chambre partagée)
Autre structures (chambre individuelle)
Autre structures (chambre partagée)
Appartement personnel
Pour combien de nuits environ? :________________________________

Suivi des recommandations COVID-19

Respect du confinement (pendant la période de confinement, du 16 mars au 11 mai)

La personne a-t-elle pu avoir une chambre individuelle (ou en couple ou famille) pendant le confinement? Oui / Non
Si non, pourquoi? Pas de chambre individuelle proposée ou disponible / Refus de la personne / Autre
Le confinement a-t-il été respecté (sorties limitées, moins d'1km, contacts < 5/jour) ? plutôt oui / plutôt non
Le confinement a-t-il été compris? plutôt oui / plutôt non

Respect des mesures barrière (pendant la période de confinement, du 16 mars au 11 mai)

Application du lavage des mains plusieurs fois par jour et/ou solutions hydro-alcooliques ?plutôt oui / plutôt non
Si plutôt non, pourquoi ? Pas de savon / Pas de solutions hydro-alcooliques / Accès difficile à un point d'eau / Difficultés de compréhension / Ne se sent pas concerné
Application de la distanciation sociale > 1mètre ? plutôt oui / plutôt non
Si plutôt non, pourquoi ? Difficultés de compréhension / Ne se sent pas concerné /Distanciation impossible (vie collective) / Distanciation culturellement inconcevable / Autre
Application du port d'un masque ? plutôt oui / plutôt non
Si plutôt non, pourquoi ? Masques non fournis / Masques non supportés / Difficultés de compréhension /Refus, ne se sent pas concerné
